# Supplementary material for: Emission of floral volatiles is facilitated by cell-wall non-specific lipid transfer proteins
Source: Nat Commun. 2023 Jan 19;14:330. doi: 10.1038/s41467-023-36027-9 (PMC9852552; doi:10.1038/s41467-023-36027-9)
Supplement: Supplementary file 2 — Description of Additional Supplementary Files [file 41467_2023_36027_MOESM2_ESM.pdf]

Description of Additional Supplementary Files:

Supplementary Data 1: Sequences of mature nsLTP proteins used for the phylogenetic analysis presented in Supplementary Figure 1.

Supplementary Data 2: Sequences of mature nsLTP proteins used for the phylogenetic analysis presented in Figure 1b.
