# Supplementary material for: Emission of floral volatiles is facilitated by cell-wall non-specific lipid transfer proteins
Source: Nat Commun. 2023 Jan 19;14:330. doi: 10.1038/s41467-023-36027-9 (PMC9852552; doi:10.1038/s41467-023-36027-9)
Supplement: Supplementary file 3 — Supplementary Data 1 [file 41467_2023_36027_MOESM3_ESM.pdf]

Supplementary Data 1: Sequences of mature nsLTP proteins used for the phylogenetic analysis presented in Supplementary Figure 1.

>AtLTP1.1
ALSCGEVNSNLKPCTGYLTNGGITSPGPQCCNGVRKLNGMVLLDRRQACRCIKNAARNPGLNADR
AAGIPRRCGIKIPYSIRFNTKCNTY
>AtLTP1.10
AISCNAVQANLYPCVVYVVQGGAIPYSCCNGIRMLSKQATSDKQGVCRCIKSVVGRSSIYLKKAAALP
GKCGVKLPYKIDPSTNCNSI
>AtLTP1.11
AITCGTVASSLSPCLGYLSKGGVVPPPCCAGVKKLNGMAQPDRQQACRCLQSAAKGVNPSLASGLP
GKCGVSIPYPISTSTNCATI
>AtLTP1.12
AISCGTVAGSLAPCATYLSKGGLVPPSCCAGVKTLNSMAKPDRQQACRCIQSTAKSSGLNPSLASGLP
GKCGVSIPYPISMSTNCNNI
>AtLTP1.3
AISCSVVLQDLQPCVSYLTSGSGNPPETCCDGVKSLAAATTADKKAACQCIKSVANSTVKPELAQALA
SNCGASLPVDASPTVDCTTV
>AtLTP1.4
LMSCGTVNGNLAGCIAYLTRGAPLTQGCCNGVTNLKNMASPDRQQACRCLQSAAKAPGLNTARA
AGLPSACKVNIPYKISASTNCNTV
>AtLTP1.5
ALSCGSVNSNLAACIGYVLQGGVIPPACCSGVKNLNSIAKPDRQQACNCIQGAARASGLNAGRAAG
IPKACGVNIPYKISTSTNCKTV
>AtLTP1.6
AVSCNTVIADLYPCLSYVTQGGPVPTLCCNGLTTLKSQAQVDRQGVCRCIKSAIGGSPRTIQNALELP
SKCGVDLPYKFSPSTDCDSI
>AtLTP1.7
TIQCGTVTSTLAQCLTYLTNSGPLPSQCCVGVKSLYQLAQPDRKQVCECLKLAGKEKGLNTDLVAALP
TTCGVSIPYPISFSTNCDSI
>AtLTP1.8
AISCGAVTGSLGQCYNYLTRGGFIPRGCCSGVQRLNSLARRDRQQACRCIQGAARASRLNAGRAAR
LPGACRVRISYPISARTNCNTV
>AtLTP1.9
IACPQVNMYLAQCLPYLKAGGNPSPMCCNGLNSLKAAAPADRQVACNCLKSVANTGINDDFAKQL
PAKCGVNIGVPFSKTVDCNSI
>AtLTP2.1
KVACIVTDLQVCLSALETPPPSAECCKNLKIQKSCLCDYMENKYLEPARKVFAACGMPYPRE
>AtLTP2.10
KAACIVTNLMSCLPAILKGSQPPAYCCEMLKEQQSCLCGYIKSHYVIPQNAHKLLAACGILYPKC
>AtLTP2.11
NVTCDATQLSSCVTAVSTGAPPSTDCCGKLKEHETCLCTYIQNSYVTSPNARKTLAACDVAYPTC
>AtLTP2.12
DVTCDAVQLSSCATPMLTGVPPSTECCGKLKEQQPCFCTYIKDQYVGSANAKKTLATCGVPYPTC

>AtLTP2.13
TQSCVPMELMPCLPAMTKREQPTKDCCENLIKQKTCLCDYIKNMFTISLVARKVLETCNVPYTSC
>AtLTP2.14
SSSCIPTELMPCLPAMTTGGQPTKDCCDKLIEQKECLCGYINNTFVSSPVARKVLEVCNIPYPSC
>AtLTP2.2
KATCDFTKFQVCKPEIITGSPPSEECCEKLKEQQSCLCAYLISQYIGNAKRVIRACGIPFPNCS
>AtLTP2.3
KVACTVTELQPCLPSVIDGSQPSTQCCEKLKEQNSCFCDYLQNQYITAAKQILAACKIPYPNC
>AtLTP2.4
GVTCSPMQLASCAAAMTSSSPPSEACCTKLREQQPCLCGYMRNQYVSSPNARKVSNSCKIPSPSC
>AtLTP2.5
GVTCDARQLQPCLAAITGGGQPSGACCAKLTEQQSCLCGFAKNQYISSPNARKVLLACNVAYPTC
>AtLTP2.6
VDPCNPAQLSPCLETIMKGSEPSDLCCSKVKEQQHCICQYLKNSFLNSPNAKIIATDCHCPYPKC
>AtLTP2.7
KVVCIVTDLRVCLPAVEAGSQPSVQCCGKLKEQLSCLCGYLKIQYVSSGKAQKVLTACAIPIPKC
>AtLTP2.9
AVTCSPMQLSPCATAITSSSPPSALCCAKLKEQRPCLCGYMRNRFVSTPNARKVSKSCKLPIPRC
>AtLTPc1
QQCRDELSNVQVCAPLLLPGAVNPAANSNCCAALQATNKDCLCNALRAATTLTSLCNLPSFDCGIS
>AtLTPc3
AQECGNDLANVQVCAAMVLPGSGRPNSECCAALQSTNRDCLCNALRAATSLPSLCNLPPVDCGIN
>AtLTPd1
IDLCGMSQDELNECKPAVSKENPTSPSQPCCTALQHADFACLCGYKNSPWLGSFGVDPELASALPK
QCGLANAPTC
>AtLTPd10
QGTCQGDIEGLMKECAVYVQRPGPKVSEACCRVVKRSDIPCACGRITASVIDMDKVVHVTAFCGKPL
AHGTKCGS
>AtLTPd11
AQVCGANLSGLMNECQRYVSNAGPNSPSRSCCALIRPIDVPCACRYVSRDVIDMDKVVYVARSCGK
KIPSGYKCGSK
>AtLTPd12
GERCNDSGIEVLRGCPDSIDKELPTPPSQGCCTLVRIIGMECVCEVINKEIIDMQKLVNVAAACGRPLA
PGSQCGS
>AtLTPd2
IDLCGMTQAELNECLPAVSKNNPTSPSLLCCNALKHADYTCLCGYKNSPWLGSFGVDPKLASSLPKE
CDLTNAPTC
>AtLTPd3
MSICDMDINDMQKCRPAITGNNPPPPVNDCCVVVRKANFECLCRFKFYLPILRIDPSKVVALVAKCG
VTTVPRSCQV
>AtLTPd4
IPVCNIDTNDLAKCRPAVTGNNPPPPGPDCCAVARVANLQCLCPYKPYLPTVGIDPSRVRPLLANCG
VNSPSCF
>AtLTPd5

ITACNINANHLEKCRPAVIGDNPPSPIKECCELLQAANLKCICRFKSVLPVLAVYPSKVQALLSKCGLTTI
PPACQAL
>AtLTPd9
RKGCYDLGITVLMGCPDSIDKKLPAPPSEGCCTLVRTIGMKCVCEIVNKKIIDMQKLVNVAAACGRPL
APGSQCGS
>AtLTPg1
QAGCRDTLTSLSPCLYYLNGGSSSPSWSCCRQFSTVVQSSPECLCSVVNSESSFYGFKFNRTLALNLPT
ACNVQTPSPSLCNTG
>AtLTPg10
PATCASRLLSLAPCGPFVQGFAQLPAQPCCDSLNQIYSQEATCLCLFLNNTLSFPINQTLALQLPPLCN
IPANSSTCSSS
>AtLTPg11
AVDCSSLILNMADCLSFVTSGSTVVKPEGTCCSGLKTVVRTGPECLCEAFKNSLGLTLDLSKAASLPSV
CKVAAPPSARCGLS
>AtLTPg12
PVNCLVSMLNVSDCFSYVQVGSNEIKPEAACCPELAGMVQSSPECVCNLYGGSPRFGVKLDKQRAE
QLSTICGVKAPSPSLCSVL
>AtLTPg13
KEECTAQLVGMATCLPYVQGKAKSPTPDCCSGLKQVINSDMKCLCMIIQENDPDLGLQVNVSLALAL
PSVCHATADITKCPAL
>AtLTPg14
KEECTEQLVGMATCLPYVQGQAKSPTPDCCSGLKQVLNSNKKCLCVIIQDNDPDLGLQINVSLALAL
PSVCHAAADVTKCPAL
>AtLTPg15
SSSCVSTLTTLSPCLSYITGNSTTPSQPCCSRLDSVIKSSPQCICSAVNSIPNIGLNINRTQALQLPNACN
IQTPPLTQCNAA
>AtLTPg16
STPCTSSMISTFTPCLNFITGSSGTPTAGCCDSLKTLTNTGMGCACLILTAVPLPTGFINRTLALALPRAC
KMGGVPIQCQAA
>AtLTPg19
QSSCTNALISMSPCLNYITGNSTSPNQQCCNQLSRVVQSSPDCLCQVLNGGSQLGINVNQTQALGL
PRACNVQTPPVSRCNTG
>AtLTPg2
NTPCSPSMLSSVTGCTSFLTGGFPTSDCCGALKSLTGTGMDCLCLIVTAVPISIPINRTLAISLPRACGIP
GVPVQCKAS
>AtLTPg20
LSQCTPSMMTTVSPCMGFITNSSSSPSSDCCNSLRSLTTGGMGCLCLIVTGVPFNIPINRTTAVSLPRA
CNMPRVPLQCQAN
>AtLTPg21
APDCMANLMNMTGCLSYVTVGEGGAAKPDKTCCPALAGLVESSPQCLCYLLSGAAQLGIKIDKAKA
LKLPGVCGVITPDPSLCSLF
>AtLTPg22
IKGCQDAMSDLYSCLPFVTNKAKAPDSTCCSTLKVKIDKGQTRKCLCTLVKDDDPGLGFKVDANRAM
SLPSACHVPANISQCPDL

>AtLTPg23
SSDCSTVIYSMMDCLGYLGVGSNETKPEKSCCTGIETVLQYNPQCICAGLVSAGEMGIELNSTRALAT
PKACKLSIAPPHCGII
>AtLTPg26
QSSCTNVLISMAPCLSFITQNTSLPSQQCCNQLAHVVRYSSECLCQVLDGGSQLGINVNETQALALP
KACHVE
>AtLTPg29
ASDCDTLVITLFPCLPFISIGGTADTPTASCCSSLKNILDTKPICLCEGLKKLGIKLNVTKSATLPVACKLNA
PPVSACDSL
>AtLTPg31
SVDCSTLILNMADCLSFVSSGGTVAKPEGTCCSGLKTVLKADSQCLCEAFKSSLGVTLNITKASTLPAA
CKLHAPSIATCGLS
>AtLTPg5
STDCSSVIYSMVDCLSFLTVGSTDPSPTKTCCVGVKTVLNYSPKCLCSALESSREMGFVLDDTKALAMP
KICNVPIDPNCDVS
>AtLTPg6
REECTNQLIELSTCIPYVGGDAKAPTKDCCAGFGQVIRKSEKCVCILVRDDDPQLGIKINATLAAHLPSA
CHITAPNITDCISI
>OsLTP1.1
AVQCGQVMQLMAPCMPYLAGAPGMTPYGICCDSLGVLNRMAPADRVAVCNCVKDAAAGPAVD
FSRASALPAACGLSISFTIAPNMDCNQV
>OsLTP1.10
AITCGQVNSAVGPCLTYARGGAGPSAACCSGVRSLKAAASADRRTACNCLKNAARKGLNAGNAASI
PSKCGVSVPYTISASIDCSRV
>OsLTP1.12
AITCGQVGSAIAPCISYVTGRGGLTQGCCNGVKGLNNAARADRQAACRCLKTLAGKSLNLGAAAGIP
GKCGVNVGFPISLSTDCSKV
>OsLTP1.13
AITCGQVGSAIAPCISYVTGRSGLTQGCCNGVKGLNNAARADRQAACRCLKSLAGKSLNLGTVAGV
PGKCGVNVGFPISLSTDCNKV
>OsLTP1.14
ITCGQVNSAVGPCLTYARGGGAGPSAACCNGVRSLKSAARADRRTACNCLKNAARKGLNAGNAAS
IPSKCGVSVPYTISASIDCSRV
>OsLTP1.15
AVTCGQVVSMLAPCIMYATGRVSAPTGGCCDGVRTLNSAAAADRQTTCACLKQQTSGGLRPDLVA
GIPSKCGVNIPYAISPSTDCSRV
>OsLTP1.16
AVSCGDVTSSIAPCLSYVMGRESSPSSSCCSGVRTLNGKASADRRTACSCLKNMASRNLNMGNAASI
PSKCGVSVAFPISTSVDCSKI
>OsLTP1.17
ISCGQVNSAVSPCLSYARGGSGPSAACCSGVRSLNSAATADRRTACNCLKNVAGSGLNAGNAASIP
SKCGVSIPYTISPSIDCSSV
>OsLTP1.18
ITCGQVNSAVGPCLTYARGGAGPSAACCSGVRSLKAAASADRRTACNCLKNAARKGLNAGNAASIP

SKCGVSVPYTISASIDCSRV
>OsLTP1.19
AITCGQVGSAIAPCISYVTGRGGLTQGCCNGVKGLNNAARADRQAACRCLKTLAGKSLNLGAAAGIP
GKCGVNVGFPISLSTDCRSA
>OsLTP1.2
AISCSAVYNTLMPCLPYVQAGGTVPRACCGGIQSLLAAANPDRRTICGCLKNVANGGPYITRAAALP
SKCNVSLPYKISTSVNCNAI
>OsLTP1.20
AITCGQVGSAIAPCISYVTGRSGLTQGCCNGVKGLNNAARADRQAACRCLKSLAGKSLNLGTVAGV
PGKCGVNVGFPISLSTDCNKV
>OsLTP1.3
VSCGDAVSALAPCGPFLLGGAARPGDRCCGGARALRGMAGEARRALCRCLEQSGPSFGVLPDRARR
LPALCKLGLAIPVGAATDCSKI
>OsLTP1.4
ALSCSTVYNTLLPCLPYVQSGGAVPAACCGGIRSVVAAARADRRAACTCLKNVAAGGPYISRAAGLP
GRCGVSVPFKISPNVNCNAV
>OsLTP1.6
SVSCSDVVADVTPCLGFLQGDDDHPSGECCDGLSGLVAAAAEDRQAACECLKSAVSGTAVEAAPAR
DLPADCGLSLPYTFSPDVDCSQS
>OsLTP1.7
AVTCGDVDASLLPCVAYLTGKAAAPSGDCCAGVRHLRTLPVAERRFACDCVKKAAAKGLNGDAIRD
LPAKCAAPLPFPLSLDFDCNTI
>OsLTP1.8
VTCGQVVSMLAPCIMYATGRVSAPTGGCCDGVRTLNSAAAADRQTTCACLKQQTSGGLRPDLVAG
IPSKCDVNIPYAISPSTDCSRV
>OsLTP1.9
AVSCGDVTSSIAPCLSYVMGRESSPSSSCCSGVRTLNGKASADRRTACSCLKNMASRNLNMGNAASI
PSKCGVSVAFPISTSVDCSKI
>OsLTP2.1
ATKCDPLALRPCAAAILWGEAPSTACCAGLRAQKRCLCRYAKNKYINSQNSRKVAAACSVPAPRC
>OsLTP2.10
PVQCDPGKLSACAVPIFFGTAPSKSCCSNLRAQEDGCFCQYARDSYINSTNARNTIAACGIAFPSC
>OsLTP2.11
AAQCDPEQLSACVSPIFYGTAPSESCCSNLRAQQEGCLCQYAKDSYVNNTNARKTIAACGIPIPSC
>OsLTP2.12
TAQCNAGQLAICAGAIIGGSTPSASCCSNLRAQRGCFCQYARNSYINSANARKTLTSCGIAIPRC
>OsLTP2.13
PSRCNPTLLTPCAGPALFGGPVPPACCAQLRAQAACLCAYARSSYIRSPNARRLFAVCGLPMPQCS
>OsLTP2.2
KASCDLMQLSPCVSAFSGVGQGSPSSACCSKLKAQGSSCLCLYKDDRIVSSNRTKRVFTACKVPAPN
C
>OsLTP2.3
GAGCNAGQLTVCTGAIAGGARPTAACCSSLRAQQGCFCQFAKDRYVNSPNARKAVSSCGIALPTCH
>OsLTP2.4

EAACDALQLSPCASAIIGNASPSASCCSRMKEQQPCLCQYARDRYVNSPNGKKVLAACHVPVPSC
>OsLTP2.5
AATCTPTQLTPCAPAIVGNSPPTAACCGKLKAHPASCFCQYKKDKYVNSPNGKKVFATCKVPLPKC
>OsLTP2.6
AAGCNPSALSPCMSAIMLGAAPSPGCCVQLRAQQPCLCQYARDSYVTSPSAQRAVKACNVKANC
>OsLTP2.7
PPQCDPGLLSPCAAPIFFGTAPSASCCSSLKAQQGCFCQYAKDSYINSTNARKMIAACGIPLPNCG
>OsLTP2.8
PPQCDPGLLSPCAAPIFFGTAPSASCCSSLKAQQGCFCQYAKDSYINSTNARKMIAACGIPLPNCG
>OsLTP2.9
LVVCDPGLLSPCAAPIFFGTAPSASCCSSLKAQQGCFCQYAKDSYINSTNARKMIAACGIPFPNCS
>OsLTPc1
GGECVPQLNRLLACRAYAVPGAGDPSAECCSALSSISQGCACSAISIMNSLPSRCHLSQINCSA
>OsLTPc2
QPSCAAQLTQLAPCARVGVAPAPGQPLPPAECCSALGAVSHDCACGTLDIINSLPAKCGLPRVTCH
>OsLTPd1
FMVCGVDADRMAADCGSYCRAGSRERPRRECCDAVRGADFKCLCKYRDELRVMGNIDAARAMQI
PSKCRIKGAPKSC
>OsLTPd10
DEGCSRDLQDLIMECQKYVMNPANPKISNACCSVIQKANVPCLCSKVTKEIVCMEKVVYVADYCKKP
LQPGSKCGR
>OsLTPd12
ADKCEKDLDLLMGSCEGYLRFPAEAKASRACCGAVRRVDVGCLCGMVTPEVVCMDKAVYVAAYCH
RPLLPGSYCGS
>OsLTPd2
LSMCGVDRSAVALCRSYCTVGSAEKPTKECCKAVANADFQCLCDRRDMLRNLENIDADRATQIPSK
CGVPGASSSCK
>OsLTPd3
RAVCNMSNDEFMKCQPAAAATSNPTTPSAGCCSALSHADLNCLCSYKNSPWLSIYNIDPNRAMQL
PAKCGLTMPANC
>OsLTPd4
HGICNLSDAGLQACKPAAAVRNPADPSSECCDALAAADLPCLCRYKGSAGWVRFYGIDLNRAMTLP
GKCGLTLPAHC
>OsLTPd9
SGDCSSDVQDLMANCQDYVMFPADPKISQACCAAVQRANMPCVCNKVIPEVICMDKVVYVVAFC
KKPFQPGSNCGS
>OsLTPg1
SPACMDAVLNMSDCLTYVMNGSTARKPDEPCCPELAGLLESKPVCLCQLLAGSSYDISVDYKRAMAL
PGICGLAAPPVTACALL
>OsLTPg10
KAECADKLMALATCLTYVEEKATAPTRDCCAGLGQVVAGSKKCLCVLVKDDEPALGFRINVTRAMDL
PSGCSIAATFSDCPKM
>OsLTPg12
DQACLTALLNMSDCLTYVQNGSRARRPDKPCCPELAGLVESNPVCLCELLSGDSYGIAVDYSRALAL

PAICRVSTPPVSTCAAF
>OsLTPg14
SGSCMTEIISLASCLGYMSGNSSAPKPSCCTALSSVVTSKPACLCAVLGGASSLGVTINNTRALELPAA
CNVKTPPASQCSTV
>OsLTPg15
NNGCSSVMMTLSPCLDYISGKSPIPEFTCCTTLAGVVQSDPRCLCMVLDSAASFGISINHTRALELPGV
CKVQAPPISQCTAV
>OsLTPg16
QSGCTSEMVSLAPCLDYMQGNASRPTASCCAALSSVVKSRPECLCAVLGGASSLGVTVNTTRALELP
AACGVKTPPPSECSKV
>OsLTPg17
AASCTASLITSFTPCFNFITSSSGGGAPTAECCQSVAAMINTSASCACLVLTGVPLGIPINRTLAVTLPKA
CNSMSVPLQCKDT
>OsLTPg18
TSGCTQTLLSMSPCLNYLTGNETAPSASCCGKLGEVVKSQPECLCVALNATAALGLSINRTRALGLPD
ACKVQTPPVSNCKSG
>OsLTPg19
RAECADKLMALSTCLTFVQDGASGAPTPDCCSGLKAVLAASRKCLCVLIKDDDPNLGLKINVTKALSL
PQLCNAPANISDCPRL
>OsLTPg2
RTECADQLVGLAPCLQYVQGEAKAPAPDCCGGLRQVLGKSPKCLCVLVKDDDPNLGIKINATLALAL
PSACGATHANVSHCPQL
>OsLTPg20
SGACTAVLAKLADCVQYATAGSPLRQPPGSCCTEVERGVKDPAAVGCVCTLLAGNTYGLPLNLTRAA
GLPAACGAPPTALSNCNVP
>OsLTPg21
ASDCGSSITALAGCLTYITPGSPEARPAKDCCAGVKSALGSPAAVACLCGALGQDFGIKINYTRAAALP
AACGGDSSALSKCNKK
>OsLTPg25
ATTCVASLLELSPCLPFFKDKAATAAPEGCCAGLSSIVKGEAVCLCHIVNHTLEAIGVDIPVDRAFALLR
DVCRLSPPADIISTCA
>OsLTPg3
AADCTDALLSLAGCLSYVQEGSTVAKPDAPCCSGLKGVVKKEVACLCQAFQGNFGVTLNMTKALQL
PAACKVKTPPFSKCHLS
>OsLTPg4
ELDCTGALLNLSSCLTYVEYRSTLTRPDKGCCGALAGVVDGEAACLCGLVGGAYGVRVDAVRALALP
TICRVDAPPPRLCAAL
>OsLTPg5
AVDCTAAEALKVGACLDYVTPGNPPRSQPSKACCGEVKGVLKDIAGVGCLCAAISTHALPLPINATRV
LHLPAACGADASAFTMCLGQ
>OsLTPg6
AVDCTAEALKLADCLDYVTPGKTAPSRPSKLCCGEVKGALKDSAAVGCLCAAFTSKTLPLPINITRALH
LPAACGADASAFSKCLAP
>OsLTPg7

QTGCTAALINLYPCLNYISGNETSPTRTCCSQLATVVQSQPQCLCAAISGSSSSIGGVTIDKTRALELPK
ACNVVTPPASRCNSA
>OsLTPg8
ATSCTASLITTFTPCLNFVTGSTNGGSPTQQCCGSLAEMVRSSADCACLILTGVPFSLPINRNLAISLTK
LCNSMSVPLQCRDT
>OsLTPg9
GSGCMPELVSLSPCMGYMSGNATAPAAACCSALSGVLRSSPRCLCMVLGTAASLGVAVDTARAALL
PGACSVQAPPASQCNAA
>Ph19536
AMTCGQVQVGVVSCLPYLTNHGPLGSCCSVTKGLVTAAKTPQDRRTACACVKSALNTIKGIDLSKAA
GLSDVCHANIPFKPSLSTDCSRVN
>Ph18755
LTCGQVTSNLAPCLPYLRNTGSLGGCCGGVKGLVNAAKSKQDRQTACGCLQQAAKSIKGINLSKAA
GLPSTCGVNIPYPISPSTDCSKVQ
>Ph18086
ATITCSTVYSGLEPCLNYVVGGGKVPSECCNGLKSLLGSARTTKDLQRACYCVKSVASSVTGAQISRA
ASIPGICKARIPFKISPDVDCSKIK
>Ph25985
INCGQVDATLVPCVPYLTQGGTPASPCCDGVKRLIQMTPTQQDRQDACECVKAAAARYTNLKPDA
ATNLPSRCGVTTNIPISATTNCKSIP
>Ph36151
AIPCGTVDMKAAACISYATGKDAKPSAPCCSGLQQLAQSVKSVDDKKAICRCLKAGVKNFAGVQDK
FLSQLPTACKIKVGFPVSMNTNCETIHM
>Ph3757
AISCGQVVTSLRSCIAYVKQGGALPVACCSGIKSLNSQATTTPDRQAACSCIKSAAGSINGINFSLASSL
PSKCGVNLPYKINPKINCSTVQ
>Ph45320
AVSCGMVATDLYPCLGYVRAGGAIPVECCSGIRSLFSAASTTSDRQTVCNCMKSAAGAISGVNLNLA
AGLPSKCGVNIPYKISPSIDCNNSLCLRANTLQKMVKLAIFIVAMCMAAMAVMLSTPHAEAAISCGQ
VVSSLEPCISYVTKGGSLPTPCCDGIKTLNSQASTTPDRQAACNCIKSAAESINGINLKTAASLPSDCGF
TKIYERRNT
>Ph33937
SNDEIQCKDIAEPMKPCVDWLTTGYKRTGVPPGACCTQMSSLNSTIAKNPDDTTAICKCVKSTISNLH
INDIVAQSLPGRCLITLPFPFTPKVKCSKIK
>Ph13380
MVVVAPHAEALTCGQVTAGLAGCLPYLQNKGPIGSCCGGVKGLLSAAKTPADRKTACTCLKSAANAI
KGINPGKAAGLPAACGVNIPYKISPSTDCSRYVICSVYYCVSFNFYDQCILI
