# Supplementary material for: Emission of floral volatiles is facilitated by cell-wall non-specific lipid transfer proteins
Source: Nat Commun. 2023 Jan 19;14:330. doi: 10.1038/s41467-023-36027-9 (PMC9852552; doi:10.1038/s41467-023-36027-9)
Supplement: Supplementary file 4 — Supplementary Data 2 [file 41467_2023_36027_MOESM4_ESM.pdf]

Supplementary Data 2: Sequences of mature nsLTP proteins used for the phylogenetic analysis presented in Figure 1b.

>PhnsLTP1
AMTCGQVQVGVVSCLPYLTNHGPLGSCCSVTKGLVTAAKTPQDRRTACACVKSALNTIKGIDLSKAA
GLSDVCHANIPFKPSLSTDCSRVN
>PhnsLTP2
LTCGQVTSNLAPCLPYLRNTGSLGGCCGGVKGLVNAAKSKQDRQTACGCLQQAAKSIKGINLSKAA
GLPSTCGVNIPYPISPSTDCSKVQ
>PhnsLTP3
ATITCSTVYSGLEPCLNYVVGGGKVPSECCNGLKSLLGSARTTKDLQRACYCVKSVASSVTGAQISRA
ASIPGICKARIPFKISPDVDCSKIK
>NtnsLTP1
LTCGQVQSSLAPCVPYLLGRGPLGGCCGGVKRLLGAARTPADRKTACNCLKSAANTFKGIDMGNAA
RLPGTCGVNIPYKISPSTDCSKVQ
>AtnsLTP2
LMSCGTVNGNLAGCIAYLTRGAPLTQGCCNGVTNLKNMASTTPDRQQACRCLQSAAKAVGPGLN
TARAAGLPSACKVNIPYKISASTNCNTVR
>AansLTP3
ISCGQVVSKLTPCLGYLQKGGPVPPACCSGVKALNDAAKSTPDRQTACTCLKNAYSANSGISSSNAA
GLPGKCGVSIPYKISPGTDCTKVQ
>AtnsLTP1
ALSCGSVNSNLAACIGYVLQGGVIPPACCSGVKNLNSIAKTTPDRQQACNCIQGAARALGSGLNAG
RAAGIPKACGVNIPYKISTSTNCKTVR
>AtnsLTP5
AISCGAVTGSLGQCYNYLTRGGFIPRGCCSGVQRLNSLARTTRDRQQACRCIQGAARALGSRLNAGR
AARLPGACRVRISYPISARTNCNTVR
>NtnsLTP4
DISCGQVVASLSPCISYVRQGGAIPAPCCSGINSLNNQATSTPDRQTACNCIKSAAAGISGINFSLAGS
LPSKCGVNLPYKISPSIDCSTVQ
>StnsLTP1
LSCGEVTSGLAPCLPYLQGRGPIGGCCGGVKGLLGAAKTPEDRKTACTCLKSAANSIKGIDTGKAAGL
PGVCGVSIPYKISPSTDCSKVQ
>NsnsLTP1-like
TAADETVTCNTVYDSLEPCLGYVLGGASVPPECCSGLKSLLGAARTRTDRQSACQCVKSVASSATGD
QISRAASIPGICKAKLPFKISANVDCSKIK
>CansLTP1-like
TTTTASSADGDATVTCSTVYSNLEPCLSYVLGGGLNVPSECCSGLKSLLSTARTKSDLQSACNCVKSV
ASRATGVQINRAAKIPGICEANIPFKISPNVDCSKIK
>StnsLTP1-like
AVSCNTVYSNLEPCLGFVLNGGPTVPSACCSGLKSLLVAAGTTADRQSACKCIKSLASSANGVQIGRA
SQLPGICNAHIPYQISPNVDCSKIT
>OmnsLTP
ISTVVVARAALSCSTVYNTLLPCLPYVQSGGAVPAACCGGIRSIVSAARTTADRRAACTCLKNVAAGA

AGGPYISRAAGLPGRCGVSVPFKISPNVNCNAIN
>Ph45320
AVSCGMVATDLYPCLGYVRAGGAIPVECCSGIRSLFSAASTTSDRQTVCNCMKSAAGAISGVNLNLA
AGLPSKCGVNIPYKISPSIDCNNSLCLRANTLQKMVKLAIFIVAMCMAAMAVMLSTPHAEAAISCGQ
VVSSLEPCISYVTKGGSLPTPCCDGIKTLNSQASTTPDRQAACNCIKSAAESINGINLKTAASLPSDCGF
TKIYERRNT
