# Supplementary material for: Emission of floral volatiles is facilitated by cell-wall non-specific lipid transfer proteins
Source: Nat Commun. 2023 Jan 19;14:330. doi: 10.1038/s41467-023-36027-9 (PMC9852552; doi:10.1038/s41467-023-36027-9)
Supplement: Supplementary file 5 — Reporting Summary [file 41467_2023_36027_MOESM5_ESM.pdf]

nature portfolio

Corresponding author(s): Natalia Dudareva

Last updated by author(s): 01/12/2023

Reporting Summary

Nature Portfolio wishes to improve the reproducibility of the work that we publish. This form provides structure for consistency and transparency in reporting. For further information on Nature Portfolio policies, see our Editorial Policies and the Editorial Policy Checklist.

Statistics

For all statistical analyses, confirm that the following items are present in the figure legend, table legend, main text, or Methods section.

n/a	Confirmed	
☐	☒	The exact sample size (*n*) for each experimental group/condition, given as a discrete number and unit of measurement
☐	☒	A statement on whether measurements were taken from distinct samples or whether the same sample was measured repeatedly
☐	☒	The statistical test(s) used AND whether they are one- or two-sided<br>*Only common tests should be described solely by name; describe more complex techniques in the Methods section.*
☒	☐	A description of all covariates tested
☒	☐	A description of any assumptions or corrections, such as tests of normality and adjustment for multiple comparisons
☐	☒	A full description of the statistical parameters including central tendency (e.g. means) or other basic estimates (e.g. regression coefficient) AND variation (e.g. standard deviation) or associated estimates of uncertainty (e.g. confidence intervals)
☐	☒	For null hypothesis testing, the test statistic (e.g. *F*, *t*, *r*) with confidence intervals, effect sizes, degrees of freedom and *P* value noted<br>*Give P values as exact values whenever suitable.*
☒	☐	For Bayesian analysis, information on the choice of priors and Markov chain Monte Carlo settings
☒	☐	For hierarchical and complex designs, identification of the appropriate level for tests and full reporting of outcomes
☒	☐	Estimates of effect sizes (e.g. Cohen's *d*, Pearson's *r*), indicating how they were calculated

Our web collection on statistics for biologists contains articles on many of the points above.

Software and code

Policy information about availability of computer code

Data collection	GC-MS data were collected and analyzed using Agilent MassHunter Workstation Software v.B. 07.01. qRT-PCR data were collected using StepOne v.2.2.2. HPLC data collection was performed using OpenLAB CDS Chemstation Rev. C.01.08[210]. Confocal images were captured with (ZEN)-Black Edition software (Zen2.6, Carl Zeiss Inc).
Data analysis	GC-MS data were analyzed using Agilent MassHunter Workstation Software v.B. 07.01, HPLC analysis was performed using Open LAB CDS Chemstation Rev. C.01.08[210], Excel 365 and GraphPad Prism version 9 were used to analyze data, MEGA 7 version 7.0.18 was used for phylogenetic analysis. Confocal microscopy images were analyzed using ZEN 2.6 lite (blue edition).

For manuscripts utilizing custom algorithms or software that are central to the research but not yet described in published literature, software must be made available to editors and reviewers. We strongly encourage code deposition in a community repository (e.g. GitHub). See the Nature Portfolio guidelines for submitting code & software for further information.

Data

Policy information about availability of data

All manuscripts must include a data availability statement. This statement should provide the following information, where applicable:

- Accession codes, unique identifiers, or web links for publicly available datasets
- A description of any restrictions on data availability
- For clinical datasets or third party data, please ensure that the statement adheres to our policy

Raw data for all Figures containing experimental data are provided in Source Data file. GeneBank/EMBL accession numbers for the sequences reported in this paper

are provided.

Human research participants

Policy information about studies involving human research participants and Sex and Gender in Research.

Reporting on sex and gender	N/A
Population characteristics	N/A
Recruitment	N/A
Ethics oversight	N/A

Note that full information on the approval of the study protocol must also be provided in the manuscript.

Field-specific reporting

Please select the one below that is the best fit for your research. If you are not sure, read the appropriate sections before making your selection.

[x] Life sciences [ ] Behavioural & social sciences [ ] Ecological, evolutionary & environmental sciences

For a reference copy of the document with all sections, see nature.com/documents/nr-reporting-summary-flat.pdf

Life sciences study design

All studies must disclose on these points even when the disclosure is negative.

Sample size	A minimum sample size of 3 independent biological experiments was chosen to obtain valid statistical analyses. A sample size of 3 was accepted to be sufficient to perform statistical tests and account for positional effect of transgene in research using transgenic plants.
Data exclusions	No data exclusion
Replication	At least three biological replicates were performed for each experiment. All attempts at replication were successful and all data included in the manuscript.
Randomization	Plants were randomized in greenhouse, and sample orders were randomized during analysis.
Blinding	Blinding is not necessary because results are quantitative for all experiments, and not subjective. Blinding was not possible in these experiments, as samples were analyzed immediately following treatments, and thus the treatment was known to the investigator.

Reporting for specific materials, systems and methods

We require information from authors about some types of materials, experimental systems and methods used in many studies. Here, indicate whether each material, system or method listed is relevant to your study. If you are not sure if a list item applies to your research, read the appropriate section before selecting a response.

Materials & experimental systems

n/a	Involved in the study	
[x]	[ ]	Antibodies
[x]	[ ]	Eukaryotic cell lines
[x]	[ ]	Palaeontology and archaeology
[x]	[ ]	Animals and other organisms
[x]	[ ]	Clinical data
[x]	[ ]	Dual use research of concern

Methods

n/a	Involved in the study	
[x]	[ ]	ChIP-seq
[x]	[ ]	Flow cytometry
[x]	[ ]	MRI-based neuroimaging
